# Tailored Surgery for Medullary Thyroid Cancer (MTC) Based on Pretherapeutic Basal Calcitonin and Intraoperative Diagnosis of Desmoplastic Stroma Reaction: A Proposal for a New Surgical Concept

**DOI:** 10.1245/s10434-025-16958-x

**Published:** 2025-03-06

**Authors:** Martin B. Niederle, Teresa Binter, Philipp Riss, Bruno Niederle, Christian Scheuba

**Affiliations:** 1 Division of Visceral Surgery, Department of General Surgery, Medical University of Vienna, Vienna, Austria; 2 Department of General Anesthesia, General Intensive Care and Pain Management, Medical University of Vienna, Vienna, Austria; 3 Division of Visceral Surgery, Department of General Surgery, Senior Clinical Investigator; Former Chief, Section Endocrine Surgery, Medical University of Vienna, Vienna, Austria

**Keywords:** Medullary thyroid carcinoma, Pretherapeutic calcitonin cutoffs, Desmoplastic stroma reaction, Tailored surgery, Risk stratification, Prognosis

## Abstract

**Background:**

Pretherapeutic sex-specific basal calcitonin (bCt) cutoff levels and intraoperative diagnosis of desmoplastic stroma reaction (DSR) by frozen section independently facilitate the prediction of lymph-node metastases (LNM) and long-term outcomes in patients with medullary thyroid cancer (MTC). The relevance of combining these two parameters to “tailor” lymph-node (LN) surgery has thus far not been analyzed.

**Patients and Methods:**

This single-center analysis included 306 patients covered by a calcitonin screening program. A uniform surgical protocol [thyroidectomy, bilateral central neck dissection (CND), lateral neck dissection (LND)] was applied. Risk groups were subdivided on the basis of predefined bCt cutoffs (“minimal risk,” “low risk,” and “high risk”), and the intraoperative classification of DSR was correlated with LN involvement in each patient. Biochemical long-term outcomes (mean follow-up: 8.3 years) were documented with the endpoints “disease-free,” “persistent,” or “recurrent” disease.

**Results:**

Patients in the “minimal risk” group (37.6%) and “low risk” group (16.3%) showed central but never lateral LNM in 2.6% and 6.0% of patients, respectively (cure rate: 98.2%). In the “high risk” group (46.1%), LNM (central and/or lateral) were found in 51.1% of the patients (cure rate: 60.9%). In all risk groups, DSR negativity (overall 20.6%) confirmed absence of LNM (cure rate:100%).

**Conclusions:**

The analysis facilitates the recommendation to individualize the extent of LND combining pretherapeutic bCt and the presence of DSR. Independent of bCt levels (risk groups), LND can be avoided following thyroidectomy in patients with DSR-negative tumors. Patients with DSR-positive tumors should undergo thyroidectomy and bilateral CND. In addition, in “high risk” patients, unilateral LND is recommended in those with bCt < 350 pg/mL. Bilateral LND should be discussed in patients with M0 status and bCt > 350 pg/mL.

**Supplementary Information:**

The online version contains supplementary material available at 10.1245/s10434-025-16958-x.

Medullary thyroid cancer (MTC) spreads to lymph nodes (LNs) at a very early stage and regardless of tumor size.^1,2^ During the workup of thyroid nodules, it should be the main focus to diagnose MTC before LN metastases (LNMs) develop because LNMs negatively influence prognosis.^3,4^ Early diagnosis of MTC is only provided by consequently measuring basal calcitonin (bCt) levels during diagnostic workup. Surgery, including stage-adapted lymph node dissection (LND), is the single curative treatment, as missing micro-LNMs results in persisting or recurrent disease, thus reducing cure rates and worsening long-term prognosis. For years, besides (total) thyroidectomy, bilateral central neck dissection (CND), unilateral neck dissection (ULND), or in selected patients, bilateral lateral neck dissection (BLND), have been recommended.^5^

It has been shown that both well-defined cutoffs for pretherapeutic bCt^6,7^ and the intraoperative distinction between desmoplastic stroma reaction (DSR)-negative and DSR-positive tumors can predict the presence of LNM.^8^ DSR-negative MTCs never present with LN metastases [or distant metastases (M1)],^8,9^ but since the majority of tumors are DSR-positive, diagnosis of DSR can only be used in approximately a quarter of patients for intraoperative decision-making in favor of or against extensive LND.^8,9^

The aim of this study was to analyze the combination of both pretherapeutic bCt and the intraoperative diagnosis of DSR in predicting LN involvement and long-term cure rates. On the basis of the results of this single-center long-term follow-up study, a proposal for a new tailored surgical concept is presented.

## Patients and Methods

Starting prospectively in 1994,^10,11^ bCt was determined routinely in all thyroid nodules during diagnostic workup [calcitonin (Ct) screening program], regardless of thyroid metabolism and routine thyroid ultrasound findings (number, size, and shape of nodules).

Prospective data collection included clinical, biochemical, and morphological data of all patients ultimately diagnosed with histologically verified MTC following the standard diagnostic and therapeutic procedures (SOPs).^7^ After excluding other well-known sources of elevated basal Ct,^12^ patients with reproducibly increased bCt became candidates for surgery, independent of the results of the routinely performed neck ultrasound. The findings of ultrasonography were not used for surgical decision-making, as previous analyses had revealed an unsatisfying sensitivity of ultrasound, particularly in detecting microMTCs and LNMs.^13^

Neither ultrasound-guided fine-needle aspiration biopsy (FNAB) nor FNAB with Ct measurements in wash-out fluids from fine-needle aspiration were performed during diagnostic workup in patients with repeatedly proven elevated bCt, as routine bCt measurements had been documented to yield higher sensitivity in diagnosing MTC compared with FNAB, especially in diagnosing small (≤ 10 mm) curable MTC.^14^

Moreover, FNAB may traumatize the tumor, potentially triggering the development of scar tissue with a misinterpretation of tumor DSR-positivity.^15^ Therefore, the indication for surgery was based on bCt values only.^7^

The consequences of treated and untreated “sporadic” hypercalcitoninemia, with the arguments for and against “active surveillance” (with postponed surgery if bCt increases over time) or “early surgery,” were discussed in detail with the patients with “mildly elevated bCt” (group 1; definition below).

Only patients who had primary surgery and full information regarding diagnostic and clinical details were included in the final analysis combining pretherapeutic bCt, intraoperative frozen section (FS) and definitive histological results of the primary tumor, the number of removed and affected LNs, and the biochemical and clinical follow-up data. Patients with reoperations were excluded.

At the time of surgery, no patient was a member of a known MTC family. Genetic background was tested in all patients during workup, yet, for organizational reasons, mainly postoperatively.^16,17^ Before surgery, however, pheochromocytoma was excluded biochemically in all patients with hypercalcitoninemia.

### Calcitonin Measurements

In terms of bCt and postoperative Ct (post-Ct) analysis, mainly the immunochemiluminescent assay (ICMA) produced by the Diagnostic Products Corporation (DPC, Los Angeles, CA, USA) and running as a fully automated test on a Siemens 2000 Immunoassay System (Siemens Health Care, Erlangen, Germany), as previously described in detail with a sensitivity of 2 pg/mL and reference values of up to 6 pg/mL for women and 8 pg/mL for men, was applied.

For shorter periods during the study, a two-site immunoradiometric assay (Cis Bio International, Gif-sur-Yvette, France) or a one-site immunochemiluminometric assay (Nichols Institute Diagnostics, San Juan Capistrano, CA, USA) was applied. Later, bCt concentrations were determined with the Elecsys^®^ human Ct assay (Roche Diagnostics, Basel, Switzerland), which is a one-step sandwich assay based on streptavidin–biotin technology. Measurements from the other assays were consequently converted to levels of the DPC/Siemens 2000 assay using the recommended appropriate conversion factors to enable a direct comparison with studies in literature.^18–20^

### Surgery

All patients were treated with a uniform surgical approach^21^ regardless of sex, thyroid metabolism, bCt, ultrasound findings, tumor size, or genetic background.

All patients underwent thyroidectomy with microdissection of both recurrent nerves and additional removal of the central lymphatic tissue on both sides [level 6; bilateral central neck dissection (BCND)].^22–24^

Intraoperative histological analyses were done routinely on fresh FS stained with hematoxylin and eosin on all macroscopically visible tumors to confirm biochemically suspected MTC.^25^

All tumors were classified intraoperatively (confirmed postoperatively) as DSR-positive or “purely” DSR-negative (in short: “DSR-negative”), as shown in a preliminary study.^9^

Basically, DSR was defined as the presence of a newly formed fibrotic (collagenous) stroma surrounding the invasive epithelial tumor cells not found in the non-neoplastic thyroid parenchyma. A tumor was considered DSR-positive whenever a dense collagen-rich stroma was observed, irrespective of the quantity and extent of stroma formation. Only tumors with a stroma composed exclusively of loose reticular fibers and vessels were classified as DSR-negative.^26^

Surgery was completed by microdissecting both lateral neck compartments (levels 2–5) and preserving the internal jugular vein and all nerves and muscles (functional BLND), if FS confirmed MTC.^22,23,27^

Transcervical or transsternal mediastinal dissection was carried out exceptionally in patients with very large LNMs in the upper thoracic outlet or mediastinum to avoid local vascular complications.

In five patients with mildly elevated bCt (minimal oncologic risk), hemithyroidectomy and unilateral CND were performed owing to a solitary benign thyroid nodule (sporadic microMTC was incidentally found postoperatively during immunohistological workup of the resected thyroid lobe beside the dominant nodule). In two patients with documented distant metastases (M1) at the time of diagnosis, thyroidectomy with BCND^28^ and extirpation of macroscopically enlarged uni- or bilateral lateral LNs was performed.

In the second step, all surgical specimens (primary and LNs) were assessed using conventional histology and immunohistochemistry on paraffin-embedded material to confirm the preliminary intraoperative histological findings, especially the subclassification of DSR.

### TNM Classification and Staging

Tumor classification and staging were performed according to the American Joint Committee on Cancer, 8th edition.^29^ No tumor grading was performed.^30^

### Outcomes

On postoperative day 1 and during follow-up, serum calcium and parathyroid hormone levels were measured in all patients to exclude permanent postoperative hypoparathyroidism.

In addition, patients were examined pre- and postoperatively by a specialist in otolaryngology and a neurologist, in patients with suspected neurological deficits.

The long-term outcome was documented both biochemically (post-Ct levels) and on clinical controls.

Post-Ct was determined after 1, 6, and 12 months and annually thereafter utill the end of each patient’s observation period [median follow-up 8.3 years (25th–75th percentile: 4.4–13.3 years)].

The endpoints were “cured” (disease-free, including “likely disease-free”), “persisting,” and “recurrent disease.”

Patients were considered permanently cured (“disease-free”) when post-Ct remained undetectable (< 2 pg/mL) and those as “likely disease-free” when post-Ct was measurable (> 2 pg/mL) but below the upper normal sex-specific Ct limits, without evidence of structural recurrence and without increase during follow-up. Patients with post-Ct higher than the sex-specific upper normal hormone levels with/without structural recurrence were classified as “persisting disease.” “Recurrence” was related to as post-Ct < 2 pg/mL during a follow-up of at least 12 months and a slight or rapid increase in Ct with/without structural recurrence thereafter.

### Statistical Analysis

The statistical analyses were performed with IBM SPSS 28.0 for Windows and Microsoft Excel for Windows version 2404.

As all continuous parameters were not normally distributed, the parameters are presented as median and interquartile range (IQR) (25th–75th percentile) or absolute range (min–max). For group comparison of continuous parameters, the Wilcoxon signed-rank test was performed. Binominal parameters are presented as absolute numbers and percentages. To compare group differences for dichotomous proportions, Fisher’s exact test (2 × 2 or *R* × 2) was conducted. A two-sided *p*-value of > 0.05 was considered as not significant.

## Results

By routinely applying Ct screening in the workup of all thyroid nodules,^11^ 324 patients were initially diagnosed and treated with MTC. Owing to impaired renal function and therefore unreliable bCt measurments, 18 patients were excluded from further analysis.

Complete data, including pretherapeutic bCt, the intraoperative and postoperative assessment of DSR, and the postoperative course of Ct (biochemical follow-up) were analyzed in 306 patients.

The subdivision of patients into oncologic risk groups based on bCt and the demographic background information are summarized in Table 1. In accordance with the predefined clinical relevant cutoffs,^6,7^ 115/306 (37.6%) female and male patients with bCt ≤ 23 pg/mL and ≤ 43 pg/mL, respectively (expected MTC incidence in female patients, 17.1%; in male patients, 37.5%; no/very low LNM risk^7^), were assigned to group 1 (minimal oncologic risk). Altogether, 50/306 (16.3%; female and male patients with bCt 24–84 pg/mL and 44–99 pg/mL, respectively) were allocated to group 2 (low oncologic risk; 100% MTC incidence, low LNM risk), and 141/306 (46.1%) female and male patients to group 3 (female and male patients with bCt ≥ 85 pg/mL and male patients ≥ 100 pg/mL, respectively; 100% MTC incidence; high oncologic risk for LN and M1).

**Table 1:** Pretherapeutic data (sex, age, basal calcitonin) and intra- and postoperative pathohistological details (tumor diameter, desmoplastic stroma reaction, multiplicity) of the study population

Risk group	Group 1	Group 2	Group 3	Total
	Minimal oncologic risk	Low oncologic risk	High oncologic risk	
Sex-specific bCt (pg/mL)	f: ≤ 23; m: ≤ 43	f: 24–84; m: 44–99	f: ≥ 85; m: ≥ 100	
***n* (%)**	115	50	141	**306** (100)
**Sex**				
f	36	39	89	164 (53.6)
m	79	11	52	142 (46.4)
**Age** (years; median,)	60 (51–67)	62 (52–70)	58 (50–68)	**60** (51–68)
**bCt** (pg/mL; median)	15 (10–20)	51 (38–66)	698 (192–1942)	**66** (18–550)
**Tumor DM** (mm, median)	2 (1–4)	5 (4–7)	18 (11–28)	**6** (2–15)
**DSR-neg.**				**63** (20.6%)
Solitary	30	15	12	57
Multiple, unilateral	2	0	0	2
Multiple, bilateral	2	2	0	4
**DSR-pos.**				**243** (79.4%)
Solitary	46	27	99	172
Multiple - unilateral	5	0	3	8
Multiple - bilateral	30	6	27	63

*f* female, *m* male, *bCt* pretherapeutic basal calcitonin, *DM* diameter, *DSR* desmoplastic stroma reaction, *neg*. negative, *pos*. positive

Intraoperative tumor discrimination by FS yielded 243/306 (79.4%) DSR-positive and 63/306 (20.6%) DSR-negative tumors (Table 2). The majority of patients with DSR-negative tumors were included in groups 1 and 2 (51/63; 81.0%), the proportion being significantly higher in those groups than in group 3 (*p* < 0.001; Table 2). Details on pathological tumor-node-metastasis (pTNM), according to the WHO classification,^29^ are provided in Table 3.

**Table 2:** Pretherapeutic basal calcitonin level (risk groups) – Desmoplastic stroma reaction - N + M classification

Risk group	Group 1	Group 2	Group 3	p (overall)	Total n (%)
	Minimal oncologic risk	Low oncologic risk	High oncologic risk		
Sex-specific bCt (pg/mL)	f: ≤23; m: ≤43	f:24-84; m: 44-99	f: ≥85; m: ≥100		
**n (%)**	115 (37.6)	50 (16.4)	141 (46.1)		306 (100)
**DSR**					
neg.	34 (29.6)	17 (34.0)	12 (8.5)	<0.001*	63 (20.6)
pos.	81 (70.4)	33 (66.0)	129 (91.5)		243 (79.4)
**N**					
0	112 (97.4)	47 (94.0)	69 (48.9)	<0.001^+^	228 (74.5)
1	3 (2.6)	3 (6.0)	72 (51.1)		78^#^ (25.4)
**M**					
0	115 (100)	50 (100)	122 (86.5)	-	287 (93.8)
1	0 (0)	0 (0)	19 (13.5)		19^~#^ (6.2)

^*^Group 1 vs Group 2: p=0.348; Group 1 vs. Group 3: p<0.001; Group 2 vs. Group 3: p<0.001

^+^Group 1 vs Group 2: p=0.258; Group 1 vs. Group 3: p<0.001; Group 2 vs. Group 3: p<0.001

^#^All patients DSR-positive; ~ all patients N1-positive

f: female; m: male; bCt: pretherapeutic basal calcitonin; DSR: desmoplastic stroma reaction; neg.: negative; pos.: positive; N: lymph node;

M: distant metastasis

**Table 3:** Pretherapeutic basal calcitonin level (groups), pTNM classification – DSR, frequency and pattern of lymph nodes and distant metastases

Risk group	Group 1					Group 2					Group 3					Total
	Minimal oncologic risk					Low oncologic risk					High oncologic risk					
Sex-specific bCt (pg/mL)	f: ≤23; m: ≤43					f:24-84; m: 44-99					f: ≥85; m: ≥100					n (%)
n (%)	115 (37.6)					50 (16.4)					141 (46.1)					306 (100)
**T**	1a	1b	2	3a/b	4a	1a	1b	2	3a/b	4a	1a	1b	2	3a/b	4a	
n/n	114/ 115	1/115	0	0	0	47/ 50	3/50	0	0	0	35/ 141	47/ 141	43/ 141	11/ 141	5/ 141	
**DSR**																
neg	34	0	0	0	0	16	1	0	0	0	3	4	5	0	0	63 (20.6)
pos	80	1	0	0	0	31	2	0	0	0	32	43	38	11	5	243 (79.4)
**N**																
1	3	0	0	0	0	3	0	0	0	0	12	26	20	9	5	78 (25.4)
1a	3	0	0	0	0	3	0	0	0	0	0	3	5*~	0	0	14* (5.7)
1b	0	0	0	0	0	0	0	0	0	0	12	23	15	9	5	64 (45.4)
**L**	0	0	0	0	0	0	0	0	0	0	5*	7	2	1	0	15*
C+UL	0	0	0	0	0	0	0	0	0	0	5^(2^*^)^	13*	9^(2^*^)^	4*	3^(3^*^)^	34 ^(10^*^)^
C+BL	0	0	0	0	0	0	0	0	0	0	2^(2^*^)^	3	4*	4^(3^*^)^	2*	15 ^(7^*^)^
**M**																
1	0	0	0	0	0	0	0	0	0	0	5^(2+)^	1	5^+^	4^(3+)^	4^+^	19 ^(7+)^ (6.2)

f: female; m: male; bCt: pretherapeutic basal calcitonin; DSR: desmoplastic stroma reaction

T: Tumor – pathological classification; 1a: T size ≤ 1 cm and intrathyroidal; 1b: T size > 1 cm ≤ 2 cm and intrathyroidal; 2: T size > 2 cm ≤ 4 cm and intrathyroidal; 3a: T size > 4 cm and intrathyroidal; 3b: T gross extrathyroidal extension (sternohyoid, sternothyroid, thyrohyoid, omohyoid muscles); 4a: T gross extrathyroidal extension (subcutaneous soft tissue, larynx, trachea, esophagus, recurrent laryngeal nerve); 4b: T gross extrathyroidal extension (prevertebral fascia) OR envading the carotid artery, mediastinal vessels

N: lymph nodes (LN) – pathologically verified; N0: no lymph node metastasis (LNM); N1a: lymph node metastasis in the central neck (C; compartment C1a and/or b - Dralle); N1b: lymph node metastasis in the lateral neck; L only: skip LNM; C+UL: central and unilateral LNM; C+BL: central and bilateral LNM; M0: no distant metastasis; M1: distant metastasis – radiologically verified; *: patient with M1 (n=19); ~: M1 – no LND performed; +: patient with C+BL (n=7)

DSR-negative tumors were intrathyroidal, encapsulated lesions without peritumoral invasion, the majority being classified as pT1a (53/63; 84.1%) and pT1b (5/63; 7.9%). Only 5/63 (7.9%) were classified as pT2 and allocated to group 3. No LNM were found in combination with DSR-negative tumors (Tables 2 and 3). All DSR-positive tumors classified as pT2, pT3a/b, or pT4a were assigned to group 3. No pT4b tumors were diagnosed.

The median bCt of DSR-positive and DSR-negative tumors was not significantly different within the risk groups [group 1, 15 versus 14 pg/mL (*p* = 0.338); group 2, 52 versus 44 pg/mL (*p* = 0.264); group 3 (M0 only), 598 versus 502 pg/mL (*p* = 0.344)].

Multifocality was documented in 71/273 (26.0%) patients with DSR-positive tumors and in 6/63 (9.5%) with DSR-negative tumors (Table 1). Of these, two patients had unilateral tumors and four had bilateral tumors (five patients with sporadic and one patient with hereditary MTC, Supplementary Table 1).

LNMs were found in 78/306 (25.5%; all with DSR-positive tumors) patients. In groups 1 and 2, patients with LNM were rare (2.6% and 6.0%, respectively) and the proportion of patients with N1 was significantly lower than in group 3 (N1, 51.1%; *p* < 0.001; Table 2). Solitary or multiple M1 were documented only in group 3 [19/306 (6.2%) patients; lung, 7; liver, 9; and/or bones, 5; Tables 2 and 3].

Table 2 documents the correlation between the pretherapeutic risk groups and T classification with the LNM pattern and frequency. Central LNMs (CLNMs; pN1a) were identified in 3/115 (2.6%) patients in group 1 and in 3/50 (6%) in group 2 (6/165; 3.6%) and all 6 patients classified as pT1a. No lateral LNMs (LLNM; N1b) were diagnosed in groups 1 or 2. In group 3, LNMs in various locations (central and lateral) and of various frequencies were found in combination with all T stages (Table 3).

**Fig. 1:**
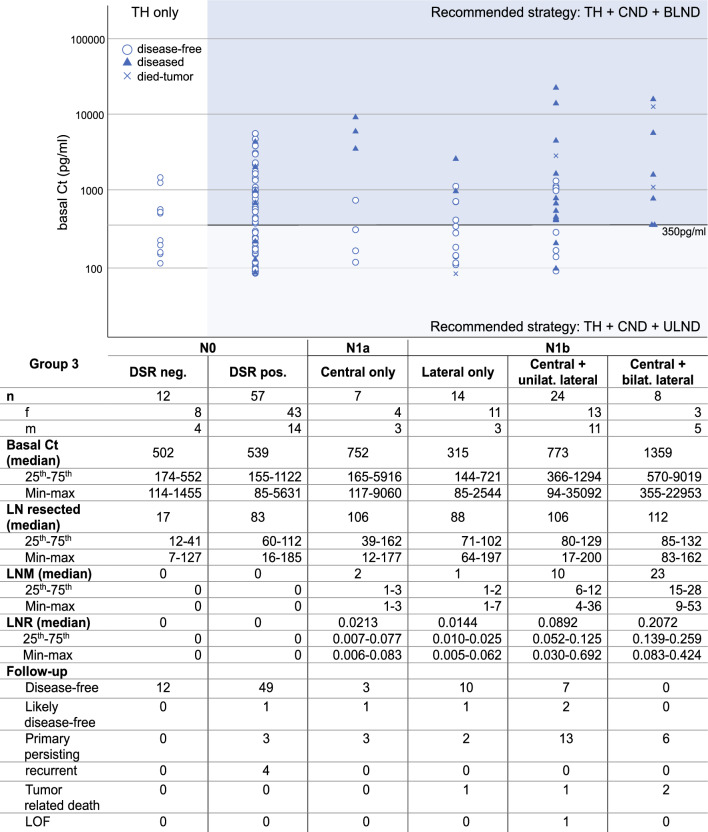
Group 3 (M0)—pretherapeutic bCt and correlation with N-classification—LNM pattern, resected LN, and follow-up; the circles/triangles/crosses represent the bCt level in each patient and the horizontal line represents bCt 350 pg/mL, the proposed cut-off level for bilateral LLNM; *DSR* desmoplastic stroma reaction, *N0* no lymph node metastasis, *N1a* central lymph node metastases, *neg*. negative, *pos*. positive, *N1b* lateral lymph node metastases, *f* female, *m* male, *Ct* calcitonin, *LN* lymph nodes, *LNM* lymph node metastases, *LNR* lymph node ratio (the number of affected LN divided by the number of resected LN), *LOF* loss of follow-up

Excluding M1 (6.2%), there was no significant difference between the bCt of patients with pN0 and pN1 in group 3 (*p* = 0.135; Fig. 1). In addition, the ranges of bCt in patients with CLNMs only (*n* = 7), LLNMs only (L-LLNM; skip; *n* = 14), CLNM+unilateral (U-LLNM (*n* = 24), and CLNM+bilateral metastases (B-LLNM; *n* = 8) overlapped broadly, thus impeding definite prediction of the presence, location, and number of LNMs in group 3 (Fig. 1). However, analyzing the subgroup of 46 patients with N1b, bCt > 350 pg/mL was identified in all 8 patients with CLNM+B-LLNM (Fig. 1).

### Outcome by Post-Ct

In total, 3 out of 306 patients (median follow-up: 100 [IQR 53–159] months) were lost to follow-up (group 1: *n* = 1; group 3:* n* = 2).

Irrespective of pretherapeutic oncologic risk groups, all 51 patients with DSR-negative tumors (all pN0) in groups 1 and 2 were cured (Table 4).

**Table 4:** All oncologic risk groups (incl. M1: n=19): DSR- + N-classification and long-term follow up

Risk group	Group 1			Group 2			Group 3		
	Minimal oncologic risk			Low oncologic risk			High oncologic risk		
Sex-specific bCt (pg/mL)	f: ≤23; m: ≤43			f: 24-84; m: 44-99			f: ≥85; m: ≥100		
n (%)	115 (37.6)			50 (16.4)			141 (46.1)		
Follow-up	N0		N1*	N0		N1*	N0		N1
	DSR neg	DSR pos		DSR neg.	DSR pos.		DSR neg.	DSR pos.	
Disease-free	34	72	2	17	29	3	12	49	20
Likely disease-free	0	5	0	0	0	0	0	1	4
Primary persistent	0	0	0	0	1	0	0	3	34^#^
Recurrent	0	0	1	0	0	0	0	4	0
Tumor-related death	0	0	0	0	0	0	0	0	12^##^
LOF	0	1	0	0	0	0	0	0	2^###^

bCt: pretherapeutic basal calcitonin; N: lymph node; N0: no lymph node metastases; N1: lymph node metastases positive.

DSR: desmoplastic stroma reaction; LOF: loss of follow-up; neg.: negative; pos.: positive;

^*^All patients N1a only (no N1b); at diagnosis M1: ^#^10/34; ^##^8/12; ^###^1/2

Overall, 108/114 (94.7%) patients in group 1 and 49/50 (98.0%) in group 2 were biochemically cured (Table 4). In group 1, mildly persisting disease (likely disease free) was identified in 5/114 (4.4%) patients and recurrent disease in 1 patient. The patient with recurrent disease had multifocal microMTCs with 1/166 positive nodes. He was biochemically disease free for 12 months (bCt < 2 pg/mL), subsequently showing a slow, step-by-step increase to 16 pg/mL within the following 140 months and remaining stable without structural recurrence for 24 months thereafter (Supplementary Table 2).

In group 2, one patient showed persisting disease, with constant bCt (8 pg/mL) during 76 months of follow-up (Supplemental Table 2).

In group 3*,* 19/141 (13.5%) patients with M1 were documented at the time of diagnosis. Out of these, 8/18 (44.4%) patients died of MTC and 10/18 (55.6%) were alive showing progressive disease [loss of follow-up (LOF), *n* = 1; Table 4].

Since palliative surgery was performed, avoiding excessive LLND in most M1 patients, these patients were excluded from further analysis. All 12 (100%) patients with DSR-negative and 69/121 (57.0%; LOF: 1) with DSR-positive tumors were cured [cure rate M0: 81/133 (60.9%)].

Classification, bCt, numbers of LNM, LN ratio (LNR; ratio of LNM to resected LNs), and follow-up data are shown in Fig. 1. In total, 8 (14.0%) out of 57 patients initially diagnosed as N0 showed biochemical evidence of persisting/recurrent disease in long-term follow-up (likely disease-free, 1; primary persisting, 3; recurrent, 4) as did 4/7 (57.1%) patients classified as pN1a (Fig. 1, Supplementary Table 2). In addition, 4 out of 14 (28.6%) patients with “skip” LNM, 16/24 (66.7%) with CLNM+U-LLNM, and none (0/8) with CLNM+B-LLNM were disease free (Fig. 1).

### Combined Analysis of bCt and Intraoperative Verification of DSR

Table 5 summarizes the correlation of bCt and DSR classification. All patients with intraoperatively documented/postoperatively confirmed DSR-negative tumors (63/285; 22.1%) were N0 and M0 and all (100%) were cured in the long-term follow-up, regardless of pretherapeutic bCt.

**Table 5:** Postoperative outcome combining preoperative bCt and intraoperative DSR and proposed surgical strategy (M0 only: *n* = 287; LOF: *n* = 2)

	Any group + DSR neg.	Group 1 or 2 + DSR pos.	Group 3 + DSR pos. + bCt ≤ 350 pg/mL	Group 3 + DSR pos. + bCt > 350 pg/mL
*n* (%)	63/285 (22.1%)	114/285 (40.0%)	41/285 (14.4%)	69/285 (24.2%)
N0	63	106	24	33
N1a	0	6	3	4
N1b unilateral	0	0	14	24
N1b bilateral	0	0	0	8
Cure rate* *n* (%)	**63/63 (100%)**	**111/113**^**+**^** (98.2%)**	**35/41 (85.3%)**	**39/68**^**+**^** (57.4%)**
Tumor-related death	0	0	1	3
**Proposed surgical strategy**	**Thyroidectomy only**	**Thyroidectomy + BCND**	**Thyroidectomy + ULND**	**Thyroidectomy + BLND**

*Patients biochemically disease-free and likely disease-free (bCt not measurable or bCt within normal range without evidence of structural recurrence/persistence); ^+^patient loss of follow-up

*DSR* desmoplastic stroma reaction, *neg*. negative, *pos*. positive, *BCND* bilateral central neck dissection, *ULND* unilateral lateral neck dissection, *BLND* bilateral lateral neck dissection

In groups 1 and 2 (minimal and low oncologic risk), only a few patients (2.6% and 6%, respectively) with DSR-positive tumors had CLNM but no LLNM, with the cure rate being 98.2% (Table 5).

After excluding M1 patients from group 3 and subdividing patients into those with bCt ≤ 350 pg/mL (*n* = 41) and > 350 pg/mL (*n* = 69; no significant sex difference in bCt; data not shown), none of the patients with DSR-positive tumors and bCt ≤ 350 pg/mL showed B-LLNM. In this subgroup, the cure rate was 35/41 (85.4%) after BLND. The cure rate was 57.4% (39/68) in patients with bCt > 350 pg/mL. None of the patients with B-LLNM, verified and followed in eight patients, were cured (Table 5).

### Complications

In accordance with the surgical SOPs of the University Hospital (a tertiary referral center), all patients received radical surgery (thyroidectomy + BCND + BLND) carried out by two experienced endocrine surgeons (C.S. and B.N.; case load > 100 procedures/year). However, various transient but no permanent complications were documented (Supplementary Table 4).

## Discussion

This is the first comprehensive analysis to combine easily available pre- and intraoperative parameters as cornerstones for a tailored surgical concept to treat biochemically diagnosed MTC.

The consideration of bCt (grouping patients by assay- and sex-specific cutoffs) and the differentiation of DSR-positive and DSR-negative tumors by intraoperative FS (including sporadic and hereditary index patients who have similar biomarker levels, considering sex, median age, tumor diameter, T classification, or the presence of M1)^6,31,32^ facilitates individual decision-making for LND without compromising cure rates. Retrospectively applying this concept, 20.6% (63/306) of the CND and 57.8% (177/306) of the LND cases could have been avoided without compromising the biochemical long-term cure rate of 96.0% (169/176).

### bCt for Surgical Decision-Making Alone

Ct is not only an established biomarker for the early diagnosis and postoperative surveillance of MTC, but also facilitates the biochemical prediction of MTC,^7^ estimation of oncologic risk at the time of diagnosis, and reliable projection of the extent of the disease, thus allowing to guide the extent of surgery.^6^

Owing to the occasionally difficult diagnosis of small primary tumors in Ct screening programs,^7^ their multifocality, and the low sensitivity of conventional imaging in diagnosing central and lateral LNMs (especially of micro-disease^13,33^), thyroidectomy and initial CLND are recommended as the initial surgical step.^34^ Dissection of the lateral compartments remains controversial and is recommended only in patients when LN involvement is suspected on preoperative imaging,^34^ as more extended surgery exposes patients to higher risks of complications.^35^ However, the sensitivity of both ultrasound and dihydroxyphenylalanine-positron emission tomography-computed tomography (DOPA-PET-CT) has been shown to be low especially for small LNM and seems to be insufficient for early diagnosis and surgery planning.^13^

To minimize surgical morbidity without compromising cure requires surgical decision-making to adapt the extent of LN surgery. bCt correlates well with tumor size and less with the degree of LNM.^32,36^ Reviewing literature,^37,38^ it is increasingly recommended to guide the extent of primary LND on the basis of bCt. However, applying different Ct assays and lack of information on sex-specific assay differences make the interpretation of published data difficult and recommendations concerning the extent of LND inaccurate.

In the current study, one of the widely used, highly sensitive ICMAs, DPC Siemens Immulite 2000, was applied to render comparisons with other reports possible.

Machens and Dralle^39^ used the same Ct assay to correlate bCt with LN involvement in the central, lateral, and mediastinal compartments of 300 patients and concludingly recommended the extent of LND on the basis of the evaluated bCt levels. Although these data derive from a single analysis only, and the recommendations do not take into account important sex-specific differences, they have nevertheless been included in various guidelines^5^ and differ in some respects with the current data.

In the current study, CLNMs but no LLNMs were documented in 2.6% and 6% in groups 1 and 2, respectively. Therefore, thyroidectomy and bilateral CND seem necessary but sufficient in all female patients with bCt up to < 85 pg/mL and male patients < 100pg/ml, respectively, to clear the central compartment of possibly microscopic, undetected disease with an excellent cure rate of 98.2%.

In contrast to Machens and Dralle,^39^ we only documented LLNMs in female and male patients starting with a bCt ≥ 85 pg/mL and ≥ 100pg/mL, respectively, regardless of the results of conventional neck imaging. Therefore, in patients with “high oncologic risk,” LND should be planned, as LLNMs may be identified in nearly half (45.4%) of the patients whose bCt levels exceed these cutoffs.

Deciding on less extensive LND seems difficult, since the bCt levels of pN0 and pN1 patients (CLNM, L-LNM, CLNM+U- and B-LLNM) were broadly overlapping and made the definite prediction of LNMs, their location, and the extent of LND impossible.

Machens and Dralle^39^ recommended BLND in patients starting with bCt > 200 pg/mL. In the current study, B-LLNMs were identified only in patients with bCt > 350 pg/mL. However, the cure rate decreased to 57.4% in patients with bCt > 350 pg/ml as compared with those with bCt ≤ 350 pg/ml (85.3%), and none of the patients with B-LLNM were cured. Poor prognosis was reported by Spannheimer et al.^40^ and Yamashita et al.^41^ in critically discussing whether LND has any positive effect in decreasing locoregional and overall recurrence or survival in MTC with LLNM.

### Combination of DSR and bCt for Surgical Decision-Making

On the basis of a preliminary study by Kaserer et al.,^42^ the clinical importance of DSR-positive and DSR-negative MTC variants was originally documented by Scheuba et al.^9^ The authors concluded that patients with the DSR-negative variant never had LNM (and M1) at the time of diagnosis or during follow-up.^8^

DSR negativity, as a specific pathohistological tumor feature included in the current WHO nomenclature,^43^ has been mentioned^15^ and confirmed independently in various clinical reports.^44,45^ Desmoplasia is accepted as a reliable and highly reproducible intraoperative marker for predicting LN involvement,^26^ and intraoperative FS can guide LN surgery primarily under collaboration with an experienced pathologist, who is aware of these two MTC variants.^25,26^

Only a “purely” DSR-negative tumor certainly lacks LNM. The strict qualitative distinction between DSR-positive and DSR-negative tumors precludes false-negative results and is a feasible and safe criterion for intraoperative decision-making regarding the necessity of LND. Quantifying DSR during surgery is not only time consuming, but also more subjective, potentially leading to less reliable results. As shown by Aubert et al.^15^ and Machens et al.,^45^ the probability of LNM rises with increasing DSR density and only “purely” DSR-negative MTC predicts LN and M negativity. Tumors with any evidence of DSR should therefore be classified as DSR-positive, not to blur the possibility of safely modifying the surgical strategy.

The DSR variants cannot be predicted either biochemically by bCt (or by CEA levels) or through pT classification (size)^8^ Overall, a quarter of MTCs within a cohort lack DSR.^9,15^ In the present cohort as well, 20.6% of the tumors were DSR-negative, and, as expected, all patients with DSR-negative tumors were biochemically cured regardless of pretherapeutic bCt and definitive pT classification.

Regardless of DSR positivity, multifocal tumors were found in one or both lobes in 67/306 (21.9%) patients. In accordance with Essig et al.,^33^ total thyroidectomy is recommended in all patients, independent of their pretherapeutic risk classification and intraoperative DSR discrimination, not to overlook bilateral multifocality. This is in contrary to the opinions of Dralle et al.^46^ and Mao et al.^47^

### Limitations and Strengths

One of the limitations of this study is the retrospective correlation of the biochemical and pathohistological data collected to propose a tailored surgical strategy that has not yet been proven prospectively.

After critical retrospective assessment, a relevant number of patients received “over-treatment,” leading to a relatively high rate of (transient) complications. However, this is a further strong argument to individualize surgery whenever possible. The initial radical surgical strategy was mainly intended to maximize cure rates at initial surgery and helped to definitively exclude micro-LNMs in all compartments. The retrospective correlation of all data serves as the basis for the present new proposal.

In addition, the number of patients with M1 at initial diagnosis was relatively low. Although various imaging techniques were applied, M1 could not be definitively excluded in all group 3 patients with relatively high bCt. This may have negatively affected the long-term results of the patients with LLNM, despite extensive LND.

The tumors were not retrospectively graded, as recommended by the WHO 2022. Grading could possibly be another helpful tool in referring to the influence of tumor aggressiveness on bCt and consequentially the surgical strategy. However, as grading is commonly unavailable pre- and intraoperatively, it may not be helpful for surgical decision-making.

The primary strength of this study is the relatively large cohort of patients diagnosed at an early stage by Ct screening, treated with a uniform surgical strategy (including radical LND in all compartments allowing correct pathohistological LN staging), and consequently, close follow-up at a single tertiary referral center.

In addition, high-quality data were provided by the prospectively created database containing all relevant clinical, biochemical, and morphological parameters, as well as long follow-up period with regular biochemical and clinical examinations.

## Conclusions

The modern management of MTC is evolving into an individualized surgical approach with intraoperative decision-making for LND “on demand,” thereby reducing surgical risk without compromising the chance of long-term cure.

The basis underlying individualized tailored surgery may be the knowledge regarding pretherapeutic bCt (made possible within “Ct screening programs”), which allows individual oncologic risk to be assessed together with the specific tumor feature (DSR) distinguished intraoperatively by FS, facilitating the detailed planning of simple thyroidectomy with/without additional LND.

**Fig. 2:**
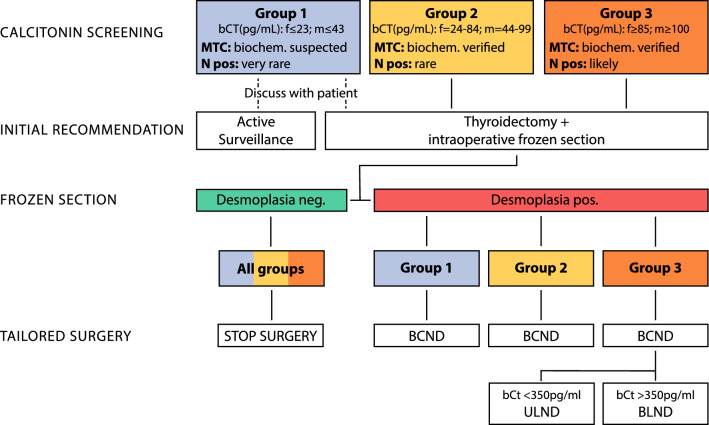
Proposal of a new surgical concept based on pretherapeutic bCt values and intraoperative morphology defined by frozen sections; *group 1* minimal oncologic risk, *group 2* low oncologic risk, *group 3* high oncologic risk, *bCt* basal calcitonin level in pg/mL, *DSR* desmoplastic stroma reaction, *neg*. negative, *pos*. positive, *BCND* bilateral central neck dissection, *ULND* unilateral lateral neck dissection, *BLND* bilateral lateral neck dissection

Figure 2 demonstrates a proposal for a modified surgical decision-making algorithm that takes these two parameters into account.

In the group 1 patients (by definition, with mildly elevated bCt), the expected MTC incidence was 17.1% in female patients and 37.5% in male patients^7^ with a very low risk of CLNM (2.6%). Therefore, the minimal oncologic risk and inconvenience of long-term active surveillance must be balanced against the potential morbidity of possibly unnecessary thyroid surgery. If the patient requests surgery even after being informed about the minor long-term consequences of mildly elevated bCt, simple thyroidectomy is the first surgical step to avoid overlooking multifocality together with FS, regardless of the genetic background.

In patients with DSR-negative tumors, who never develop LNM and M1, simple thyroidectomy, regardless of bCt, will provide cure (100%) and should be the adequate procedure in all oncologic risk groups.

Only a few patients in groups 1 and 2 with DSR-positive tumors had CLNM (2.6% and 6%, respectively) but no LLNM. Therefore, thyroidectomy with BCND should be the adequate procedure (98.2% cure rate).

CLNM and LLNM were verified in the group 3 patients. Therefore, thyroidectomy, BCND, and initial U-LLND on the side of the documented tumor may be the adequate strategy in patients with bCt < 350 pg/mL (85.3% cure rate). In the current study, all eight patients with B-LLNM (and M0) had bCt > 350 pg/mL. Therefore, thyroidectomy, BCND, and initial BLND may be recommended in this subgroup (57.4% cure rate).

## Supplementary Information

Below is the link to the electronic supplementary material.

Supplementary file1 (DOCX 44 KB)
