# Supplementary material for: Tailored Surgery for Medullary Thyroid Cancer (MTC) Based on Pretherapeutic Basal Calcitonin and Intraoperative Diagnosis of Desmoplastic Stroma Reaction: A Proposal for a New Surgical Concept
Source: Ann Surg Oncol. 2025 Mar 6;32(7):4742–53. doi: 10.1245/s10434-025-16958-x (PMC12130137; doi:10.1245/s10434-025-16958-x)
Supplement: Supplementary file 1 — Supplementary file1 (DOCX 44 KB) [file 10434_2025_16958_MOESM1_ESM.docx]

**Supplemental Table 1** Desmoplasia-negative tumors and multiplicity

| Risk  Group | | Pat. | Gender | Age | bCt  pg/mL | T | | N | | | M | CCH | PTC  pTN | FU  (mo) | bCt  (pg/mL) |  |
| --- | --- | --- | --- | --- | --- | --- | --- | --- | --- | --- | --- | --- | --- | --- | --- | --- |
|  |  |  |  |  |  | T | R/L  mm | ∑ | N1a | N1b |  |  |  |  |  |  |
| 1 | S | DN-1 | m | 57 | 9 | 1am | 0/4 | 0/13 | 0 | - | 0 | neo | - | 42 | <2 | AL |
|  | S | DN-2 | m | 67 | 10 | 1am | 0/2 | 0/2 | 0 | - | 0 | neo | 0 | 15 | <2 | AL |
|  | S | DN-3 | m | 50 | 16 | 1am | 1/1 | 0/111 | 0 | 0 | 0 | lin | 3a1b  (16/111) | 94 | <2 | AL |
|  | S | DN-4 | m | 60 | 38 | 1am | 2/1 | 0/6 | 0 | - |  | neo | - | 95 | <2 | AL |
| 2 | S | DN-5 | m | 44 | 57 | 1am | 1/1 | 0/22 | 0 | - | 0 | neo | - | 157 | <2 | AL |
|  | S | DN-6 | m | 64 | 65 | 1am | 2/2 | 0/84 | 0 | 0 | 0 | neo | - | 98 | <2 | + |

1: Group 1; 2: Group 2; S: sporadic; Pat.: identity number; Gender: m: male, f: female; Age: at diagnosis in years; bCT: basal calcitonin; T: pathological tumor classification; Tumor diameter - R: right lobe L: left lobe; N: lymph nodes, ∑ n/n: positive/extirpated nodes; N1a: central lymph nodes; N1b: lateral lymph nodes; M: distant metastasis; 0: no M; CCH: C-cell hyperplasia; neo: neoplastic; lin: linear; PTC: papillary thyroid carcinoma; FU: follow-up in months (mo); AL: alive; R: recurrence; +: tumor-unrelated death (stomach/colon cancer)

**Supplemental Table 2**: Long-term follow-up – Special clinical course of patients in Groups 1, 2 and 3 with persistent/recurrent disease (all patients DSR-positive)

| Risk  Group | | Pat. | Gender | Age | bCt  pg/mL | T | | N | | | M | CCH | PTC  pTN | FU  (mo) | bCt  pg/mL  [status]  mo |  |
| --- | --- | --- | --- | --- | --- | --- | --- | --- | --- | --- | --- | --- | --- | --- | --- | --- |
|  |  |  |  |  |  | T | R/L  mm | ∑ | N1a | N1b |  |  |  |  |  |  |
| 1 | S | 1 | m | 44 | 19 | 1am | 8/0 | 1/166 | 1/29 | R0/91  L0/46 | 0 | diff | 1am0 | 177 | 16 [R] 12 | AL |
| 2 | H | 2 | f | 73 | 73 | 1am | 4/4 | 0/186 | 0/16 | R0/61  L0/109 | 0 | neo | - | 76 | 8 [P] | + |
| 3 | H | 3 | m | 15 | 4390 | 2m | 7/25 | 0/35 | 0/35 | R0/0  L0/0 | 0 | 0 | - | 242 | 8 [R] 60 | AL |
|  | S | 4 | f | 62 | 87 | 1a | 0/9 | 0/103 | 0/10 | R0/43  L0/50 | 0 | 0 | 1a0 | 151 | 10 [P] | AL |
|  | S | 5 | f | 51 | 2056 | 1b | 14/0 | 0/98 | 0/9 | R0/42  L0/47 | 0 | 0 | - | 173 | 9 [P] | AL |
|  | S | 6 | f | 46 | 699 | 2 | 0/25 | 0/63 | 0/25 | R0/0  L0/31 | 0 | F | 1a0 | 163 | 8 [R] 60 | AL |
|  | H | 7 | f | 28 | 128 | 1am | 7/6 | 0/44 | 0/13 | R0/17  L0/15 | 0 | neo | - | 321 | 7 [R] 144 | AL |
|  | H | 8 | f | 59 | 220 | 1am | 8/9 | 0/85 | 0/14 | R0/31  L0/40 | 0 | neo | - | 178 | 7 [R] 132 | AL |
|  | H | 9 | f | 72 | 1008 | 1bm | 20/10 | 0/22 | 0/6 | R0/8  L0/8 | 0 | 0 | - | 131 | 7 [P] | + |

1: Group 1; 2: Group 2; 3: Group 3; S: sporadic; H: hereditary; Pat.: identity number; Gender: m: male, f: female; Age: at diagnosis in years; bCT: basal calcitonin;R: right; L: left; T: pathological tumor classification; Tumor diameter - R: right lobe L: left lobe; N: lymph nodes, ∑ n/n: positive/extirpated nodes; N1a: central lymph nodes; N1b: lateral lymph nodes – R: right lateral; L: left lateral; n/n: positive/extirpated nodes; M: distant metastasis; 0: no M; PTC: papillary thyroid carcinoma; FU: follow-up in months (mo); [status]: R: recurrence P: persistence; LDF: likely disease-free; number behind the status marks the months of diagnosis of recurrence; AL: alive; +: tumor-unrelated death

**Supplemental Table 3 - Complications of extended central and lateral thyroid and lymph node surgery**

| Risk group | Palsy recurrent nerve | | Hypoparathyroidism | | Others | | | Total |
| --- | --- | --- | --- | --- | --- | --- | --- | --- |
|  | Transient | Permanent/  transient | Transient | Permanent | Wound infection | Hematoma | Lymphatic  fistula | n/n (%) |
| 1 | 15 | 0 | 24 | 0 | 1 | 4 | 1 | 45/115 (39.1) |
| 2 | 2 | 0 | 13 | 0 | 1 | 2 | 1 | 19/50 (38.0) |
| 3 | 29 | 4*~/2+ | 39 | 0 | 0 | 5 | 4 | 83/141 (58.9) |
| ∑ | 46 | 6 | 76 | 0 | 2 | 11 | 6 | 147/306 (48.0) |

*: n=3: permanent vocal cord palsy following resection of the laryngeal nerve because of tumor infiltration ~ n=1: patient with transient bilateral vocal cord palsy, with one side recovering within 3 days; +: n=2 transient unilateral palsy
